# Corticosteroids versus clobazam in epileptic encephalopathy with ESES: a European multicentre randomised controlled clinical trial (RESCUE ESES*)

**DOI:** 10.1186/s13063-020-04874-2

**Published:** 2020-11-23

**Authors:** Bart van den Munckhof, Alexis Arzimanoglou, Emilio Perucca, Heleen C. van Teeseling, Frans S. S. Leijten, Kees P. J. Braun, Floor E. Jansen, on behalf of the RESCUE ESES study group, Anna Jansen, Patrick van Bogaert, Lieven Lagae, Guido Rubboli, Eija Gaily, Pierangelo Veggiotti, Gaetano Cantalupo, Giuseppe Gobbi, Dana Craiu, Petia Dimova, Thomas Bast, Julia Jacobs, Sarah von Spiczak, Anja Lübbig, Stéphane Auvin, Anne de Saint-Martin, J. Helen Cross, Richard Chin, Sameer Zuberi, Irene Garcia Morales, Georgia Ramantani

**Affiliations:** 1 Department of Paediatric Neurology, Brain Center, University Medical Center Utrecht, Member of the European Reference Network EpiCARE, Utrecht University, KC 03.063.0, PO Box 85090, 3508 AB Utrecht, The Netherlands; 2 Paediatric Clinical Epileptology, Sleep Disorders and Functional Neurology Department, University Hospitals of Lyon (HCL), Coordinator of the European Reference Network EpiCARE, Lyon Neurosciences Research Centre, Lyon, France; 3 Paediatric Epilepsy Unit, Child Neurology Department, Hospital San Juan de Dios, Universitat de Barcelona, Barcelona, Spain; 4 Clinical Pharmacology Unit, Department of Internal Medicine and Therapeutics, University of Pavia and Clinical Trial Center, IRCCS Mondino Foundation, Member of the European Reference Network EpiCARE, Pavia, Italy; 5 Department of Paediatric Neuropsychology, University Medical Center Utrecht, Utrecht University, Utrecht, The Netherlands; 6 Department of Clinical Neurophysiology, Brain Center, University Medical Center Utrecht, Utrecht University, Utrecht, The Netherlands

**Keywords:** Epilepsy, Sleep, ESES, CSWS, Corticosteroids, Clobazam, Cognition, Neuropsychology

## Abstract

**Background:**

Epileptic encephalopathy with electrical status epilepticus in sleep (ESES) is an epilepsy syndrome occurring almost exclusively in children, usually at an age between 4 and 12 years. It is characterised by abundant sleep-induced epileptic activity in the electroencephalogram (EEG) and by acquired cognitive and behavioural deficits. The goal of treatment is to prevent further decline or even improve cognitive functioning. Based on mostly small and retrospective studies, corticosteroids and clobazam are regarded by many clinicians as the most effective pharmacological treatments. This European multicentre randomised controlled trial is designed to compare the effects of corticosteroids and clobazam on cognitive functioning after 6 months. Secondary outcomes include cognitive functioning after 18 months, EEG abnormalities in sleep, safety and tolerability, and seizure frequency. We also aimed at investigating whether treatment response in epileptic encephalopathy with ESES can be predicted by measurement of inflammatory mediators and autoantibodies in serum.

**Methods:**

The pragmatic study will be performed in centres with expertise in the treatment of rare paediatric epilepsy syndromes across Europe. A total of 130 patients, 2 to 12 years of age, with epileptic encephalopathy with ESES will be enrolled and randomised in a 1:1 ratio to receive either corticosteroids (monthly intravenous methylprednisolone pulses or daily oral prednisolone) or oral clobazam for 6 months according to an open-label parallel-group design. Follow-up visits with clinical assessment, EEGs, and neuropsychological testing are scheduled for up to 18 months. Blood samples for cytokine and autoantibody testing are obtained before treatment and 8 months after treatment initiation.

**Discussion:**

The treatment of epileptic encephalopathy with ESES aims at improving cognitive outcome. This randomised controlled study will compare the most frequently used treatments, i.e. corticosteroids and clobazam. If the study proves superiority of one treatment over the other or identifies biomarkers of treatment response, results will guide clinicians in the early treatment of this severe epilepsy syndrome.

**Trial registration:**

ISRCTN, ISRCTN42686094. Registered on 24 May 2013.

The online version contains supplementary material available at 10.1186/s13063-020-04874-2.

## Background

Electrical status epilepticus in sleep (ESES) was initially described as a subclinical electroencephalographic (EEG) pattern of sleep-induced spikes and waves (SWs) in children, occupying 85–100% of slow wave sleep [1]. When bilateral ESES is associated with cognitive decline or behavioural disturbances, a diagnosis of encephalopathy with ESES can be made. The cognitive deficits are global (often referred to as continuous spikes and waves syndrome, CSWS) or confined to a specific cognitive domain (e.g. acquired aphasia or auditory agnosia in children with Landau-Kleffner syndrome, LKS) [2, 3]. These phenotypes are referred to as “typical ESES patients” [4, 5]. The exact incidence of the epileptic encephalopathy with ESES is unknown, but it is estimated to constitute 0.2–1.9% of paediatric epilepsy cases [5, 6].

ESES spectrum variants are increasingly recognised and include children with ESES and developmental delay but without arrest or regression of development [7]. Also, the spike wave index (SWI) threshold to diagnose ESES can be flexible, and can be < 85%, provided that the main feature of epileptic encephalopathy with ESES, i.e. occurrence of cognitive and behavioural deterioration associated with a striking enhancement of epileptic activity during NREM sleep, is demonstrated [8]. How ESES causes cognitive deficits and which factors determine cognitive outcome remain largely unknown. It has been suggested that ESES disrupts synaptic homeostasis, i.e. the balanced synaptic potentiation during daytime and synaptic downscaling in sleep, leading to an inefficient cerebral network [9]. Epileptic encephalopathy with ESES has been reported in patients with structural abnormalities (e.g. perinatal thalamic injury) [10, 11] and genetic disorders (e.g. a GRIN2A mutation) [12, 13], though in about half of the cases no clear cause is identified. Evidence is accumulating for a role of the immune system in patients with epilepsy [14]. In children with Landau-Kleffner syndrome, autoantibodies to central and peripheral myelin, cell nucleus, and blood vessels in sera and cerebrospinal fluid have been found [15]. In addition, in a previous study, we found that several cytokines were significantly higher in blood samples of patients with encephalopathy with ESES compared with healthy controls [16].

Whilst the EEG abnormalities characteristic of ESES generally resolve spontaneously during puberty, cognitive deficits often remain [17, 18]. Treatment during the active ESES phase may improve EEG abnormalities and daily functioning [19, 20]. In fact, successful early treatment (and thereby a shorter ESES duration) is associated with improvement of long-term cognitive outcome [18, 21]. However, the management of encephalopathy with ESES is often challenging and there is no consensus on which is the best initial treatment [22]. Moreover, no adequately powered randomised controlled trials have been performed in children with encephalopathy with ESES.

We recently performed a pooled analysis of 950 treatments in 575 cases with encephalopathy with ESES, reported in 112 articles. Conventional anti-epileptic drugs, often prescribed to control concomitantly occurring epileptic seizures, were generally not very effective in treating ESES and its associated cognitive deficits (improvement of EEG or cognition in 49%). Benzodiazepines and corticosteroids seemed to be more effective, with improvement in 68% and 81% of cases, respectively. However, in a subgroup analysis that included only consecutively assessed patients, a smaller proportion showed any improvement (34% with conventional anti-epileptic drugs, 59% with benzodiazepines, and 75% with corticosteroids). A subgroup of patients with a focal structural abnormality benefitted from epilepsy surgery. These results have to be interpreted with caution because most included studies were small, retrospective, and heterogeneous, and side effects were not considered [19].

Review articles on the treatment of encephalopathy with ESES concluded that no standard approach exists and mentioned high-dose benzodiazepines and (cortico) steroids as preferred options [5, 6, 23]. Among benzodiazepines, clobazam is often considered the most suitable although sedation and agitation are frequent side effects. Corticosteroids have been given in variable dosing regimens, and concerns for side effects such as weight gain and increased blood glucose may be a reason to consider them as a second-line option. All authors emphasise that the evidence guiding the treatment of encephalopathy with ESES is unsatisfactory, and therefore, a randomised controlled trial is urgently needed.

We hypothesise that, in comparison to treatment with clobazam, corticosteroids are more effective in improving cognitive performance and EEG abnormalities in children with encephalopathy with ESES. Based on previous studies, the difference in percentage of responders is estimated at around 25% [19]. The use of corticosteroids may be associated with more frequent or more severe side effects than clobazam.

In a multicentre randomised controlled trial with 1:1 allocation to corticosteroids and clobazam, we aim to assess whether one of the treatments is superior to the other. The following study objectives and hypotheses are addressed in a population of recently diagnosed patients with encephalopathy with ESES:

*Primary study objective*

1. To compare the effects of treatment with corticosteroids or clobazam on cognition at 6 months after start of the treatment.

*Secondary study objectives*

1. To compare the effects of treatment with corticosteroids or clobazam on sleep-induced epileptiform activity, measured as the spike wave index (SWI), in the patients’ sleep EEG.
2. To compare the effects of treatment with corticosteroids or clobazam on the frequency of any concomitant seizures.
3. To compare the side effects and tolerability of corticosteroids and clobazam.
4. To compare the effects of treatment with corticosteroids and clobazam on subjective daily functioning, as measured with a visual analogue score (VAS).
5. To assess demographic and disease-related biomarkers, including immunological biomarkers, as potential predictors of disease activity and response to treatment with corticosteroids or clobazam.

The study is conducted as a randomised open-label parallel-group controlled trial with 1:1 treatment allocation to clobazam and corticosteroid treatment arms and is aimed at proving superiority of one treatment over the other. The trial also uses a pragmatic approach, whereby participating investigators will be allowed to apply, within predetermined limits, the dosing schedules which they consider best according to their judgement and patient response. The same flexibility will also apply to the option of using i.v. versus oral steroids, which will be left to the discretion of the treating physicians.

## Methods

### Study setting

Study preparations have been initiated in 22 centres with expertise in the treatment of rare paediatric epilepsy syndromes across 12 European countries. Details of the participating centres can be found under the “Trial status” section at the end of this manuscript.

### Study population

The study will include 130 patients with encephalopathy with ESES, with typical or atypical presentation and symptoms, according to the following eligibility criteria. The overall duration of follow-up will be 18 months.

#### Inclusion criteria

- Age at inclusion, 2 up to 12 years.
- A diagnosis within 6 months prior to enrolment (preferably as close to enrolment as possible) of either:
  - Bilateral sleep-induced epileptiform activity with an SWI > 85% in non-REM sleep and developmental delay, arrest, or regression (“typical epileptic encephalopathy with ESES”).
  - Arrest or regression of development and bilateral sleep-induced epileptiform activity with an SWI > 50%, or unilateral sleep-induced epileptiform activity with an SWI > 85% in non-REM sleep (“atypical epileptic encephalopathy with ESES”).
  - Regression of development and unilateral epileptiform activity with an SWI > 50% in non-REM sleep (“atypical epileptic encephalopathy with ESES”).
- No previous treatment with either corticosteroids or clobazam.
- No current treatment, nor treatment in the previous 3 months, with carbamazepine, oxcarbazepine, vigabatrin, tiagabine, gabapentin, and pregabalin. These drugs potentially increase the SWI during sleep and may cause an electrographic pattern fulfilling the criteria for ESES and subsequently worsen outcome in children with epileptic encephalopathy with ESES and may thereby influence treatment results. Therefore, inclusion of such cases with possible “treatment-induced ESES” is not desirable.
- Written informed consent by parents/legal representatives.

#### Exclusion criteria

- Patients with an SWI during wakefulness of > 50%.
- Any condition that, in the investigator’s judgement, contraindicates the use of corticosteroids or clobazam, such as acute or chronic infectious disease (e.g. tuberculosis, HIV), immunodeficiency, severe osteopenia/osteoporosis, diabetes mellitus, Cushing syndrome, severe respiratory insufficiency, severe liver failure, or gastrointestinal ulcer.

### Informed consent

Parents/legal representatives of potentially eligible patients are informed by the treating doctor about the background, study design, and study procedures. A medical ethics committee-approved patient information leaflet describing the study background, aims, design, procedures, and timeline and highlighting any differences with standard care is provided to all parents/legal representatives. A leaflet version describing the study in basic language for the children above 6 years of age meeting their abilities to understand the content is available. Parents/legal representatives/patients have at least 7 days to consider participation, and their questions will be answered. Informed consent forms with approval for participation in the study, including the collection of blood samples and the storage of study data for a fixed period (depending on the standard per country, 15 years for the Netherlands), will be signed and filed. Study participants may withdraw from the trial anytime during the conduct of the study.

### Treatment

Patients will be enrolled by their treating physician/study doctor, who has no direct insight in the allocation mechanism of the randomisation module. Treatment is allocated according to an automatic online randomisation module with block randomisation stratified for centre to the two treatment regimens (1:1 ratio). The randomisation module was programmed by a data manager. The patients and treating physicians are not blinded for treatment allocation, as this was considered not feasible with two treatment arms that differ in their prescription form (in most centres, corticosteroids are given as intravenous monthly pulses, whilst clobazam is given as a tablet to be taken daily) and because the two treatments require different monitoring. The neuropsychological assessment and EEG assessment will be performed by personnel blinded for treatment:

- Clobazam will be administered orally and increased to a dose of 0.5 mg/kg/day within 2 weeks. If well tolerated, dosage may be increased up to 1.2 mg/kg/day (given once daily, in the evening). Treatment with a dosage of at least 0.5 mg/kg/day will be continued for 6 months and thereafter will be either continued or tapered according to the treating physician’s preference. Clobazam is defined by active substance for this study. It is prescribed as tablets, and brand names include Frisium, Onfi, and Tapclob as well as generic products.
- Corticosteroids: either intravenous methylprednisolone or oral prednisolone will be used, depending on local experience and preference.
  Intravenous methylprednisolone will be given as monthly pulses. A dosage of 20 mg/kg will be given over 30 min once a day for 3 consecutive days, every 4 weeks, for a total period of 6 months with the intention to stop thereafter.
  Oral prednisolone will be administered at an initial dosage of 2 mg/kg/day (not exceeding 60 mg/day) for 1 month, followed in the 2nd through 6th month by a dosage between 1 and 2 mg/kg/day (not exceeding 60 mg/day), according to the treating physician’s judgement. Thereafter, prednisolone will be either continued or tapered according to the treating physician’s preference.

Treatment will be continued for at least 6 months, unless informed consent is withdrawn, the patient develops intolerable adverse effects, or further cognitive regression occurs, requiring an alternative intervention in the opinion of the treating physician. If cognitive regression is observed to continue after 3 months of treatment, according to the impression of the parents or physician, switching to the other treatment arm is allowed. In patients requiring switching to an alternative treatment, an EEG will be obtained before switching.

Drug adherence to oral treatment (oral clobazam or oral prednisolone) will be optimised and monitored by instructing patients to bring their empty packages of study medication to the hospital at every scheduled visit, together with completed drug intake diaries. In addition, for patients treated with clobazam, plasma levels will be measured in blood samples collected between 1 and 3 months after enrolment and between 3 and 6 months after enrolment. For intravenous methylprednisolone, adherence will be confirmed by recording drug administration at times of hospital admission for the monthly pulses.

Concomitant medication will be allowed as long as the patient fulfils the criteria for inclusion. However, changes in concomitant medications are discouraged during the first 6 months. After assessment of the primary endpoint at 6 months, subsequent treatment strategies will be left to the clinical judgement of the treating physician.

### Outcomes

The co-primary outcome measures at 6 months will be cognitive functioning, assessed with a full neuropsychological assessment (NPA):

- Intelligence quotient (IQ), or developmental quotient (DQ), compared to baseline IQ/DQ. Improvement is defined as an increase by 10 IQ/DQ points.
- Cognitive sum score (as defined below). Improvement is defined as statistically significant when improved by at least 75% of the standard deviation (SD).

Secondary outcome measures at 6 and 18 months will include the following:

- Changes in individual absolute test results, and IQ/DQ scores, compared to baseline.
- Changes in spike wave index (SWI) during non-REM sleep, compared with baseline SWI.
- Changes in seizure frequency assessed for all reported seizure types combined, with improvement being defined as at least 50% decrease as compared with baseline.
- Changes in subjective global daily functioning assessed with a visual analogue score (VAS) of − 5 to + 5 as compared with baseline.
- Safety and tolerability, as assessed by the occurrence of adverse events.
- Change in inflammatory markers post-treatment as compared to levels prior to treatment.
- Identification of autoantibodies as potential biomarkers of disease severity (TIQ, presence of developmental regression, arrest, or delay and SWI at baseline) and treatment efficacy.

### Data collection

All patient data for the study will be recorded in the online case report form (CRF) with reference to the patient study number, in compliance with Good Clinical Practice guidelines. Data will be stored in a secure data platform, managed by an independent data manager. The forms were created by the study coordinator (BvdM) and the principal investigator (FEJ) and reviewed by the steering committee. Data quality and completeness will be checked by the study monitors, who will be granted access to the online CRF and the hospital (electronic) medical records of the included patients for the duration of their monitoring activities.

*Baseline data (t = 0 months)* will be collected before treatment initiation and include patient demographics, date of ESES diagnosis, onset of ESES, seizure type(s), anti-epileptic drug history, detailed history of psychomotor development and behaviour, estimated age at onset of developmental arrest or regression, impression of global functioning assessed with VAS score (− 5 to 5), and neurological examination. Ancillary investigations related to aetiology will be reassessed or scheduled (if not yet performed) and will include a cerebral MRI (dedicated epilepsy protocol) and genetic tests if no aetiology is known (array CGH, mutation analysis including GRIN2A gene). Metabolic screening and a cerebrospinal fluid tap will be performed if considered indicated.

### Sleep EEG at baseline and after 1, 3, 6, and 18 months

The diagnosis of encephalopathy with ESES will have to be confirmed by whole-night EEG recording prior to randomisation. Depending on the logistics of the participating centres, either sleep-deprived EEGs of at least 1 h or whole-night recordings will be considered adequate for follow-up assessments. Technical requirements for the EEG recordings are specified in an appendix to the study protocol. Clinical neurophysiologists will be blinded to the type of treatment.

For each EEG, the spike wave index (SWI) will be calculated in an epoch of 10 min (600 s) duration, starting 5 min after alpha attenuation or after sleep had clinically commenced. The number of seconds containing epileptiform discharges is divided by the total number of seconds in the epoch (600) and multiplied by 100 to reflect the SWI as a percentage.

### Neuropsychological assessment at baseline, after 6 months, and after 18 months

Depending on the age and abilities of the patient, tests will be selected from a fixed battery covering the major domains of cognition (intelligence, language, memory, attention, visuospatial functions, executive functions, as specified in an appendix of the study protocol). Administration and scoring will be conducted according to the test manuals. Individual raw test scores at baseline and at follow-up will be transformed into *z*-scores, based on the mean and SD of standard scores. As a measure of overall cognitive functioning, a cognitive sum score will be calculated, representing the mean *z*-score over the 6 domains. Neuropsychologists will be blinded to treatment.

### Cytokine profiles and autoantibodies at baseline and 8 months after start of treatment (2 months after withdrawal of study steroids, to limit the influence of a possible decrease in levels caused solely by treatment)

A snapshot of around 100 cytokines will be analysed (Luminex, X-map technology) in serum of the study participants, (a) at randomisation and (b) 8 months after start of treatment (2 months after withdrawal of corticosteroids). Screening for autoantibodies will be performed using rat brain immunohistochemistry, optimised for extracellular antigens, like NmDAR, AMPaR, GAD, GABABR, LGI1, and Caspr2 [24]. In addition, all samples will be tested by in-house cell-based assays for anti-NMDAR and anti-GlyR antibodies. Samples with positive staining, but no known antibodies, will be tested by immunocytochemistry using live hippocampal neurons. If positive, immunoprecipitation will be the first step towards antigen discovery. The laboratory analyst is blinded for treatment.

### Data collection during follow-up

Epilepsy characteristics and information on neurodevelopment, other medical history, neurological examination, concomitant medications, possible treatment emergent adverse events, and sleep EEGs will be collected 1, 3, 6, and 18 months after start of treatment. Neuropsychological assessments will be repeated only after 6 and 18 months to minimise re-test bias. After 3 months, a brief assessment of cognitive performance is performed by the clinician to detect possible ongoing regression that may warrant a change of treatment. In the corticosteroid treatment group, additional safety and tolerability assessments will include monitoring of blood pressure, glucose, and protein in urine and weight monitoring once weekly plus additionally at any hospital visits (including admissions for methylprednisolone pulses, when applicable). In case of stress, high fever or illness managements in these situations will be left to treating physician and details will be recorded in eCRF.

### Participant timeline

**Fig. 1:**
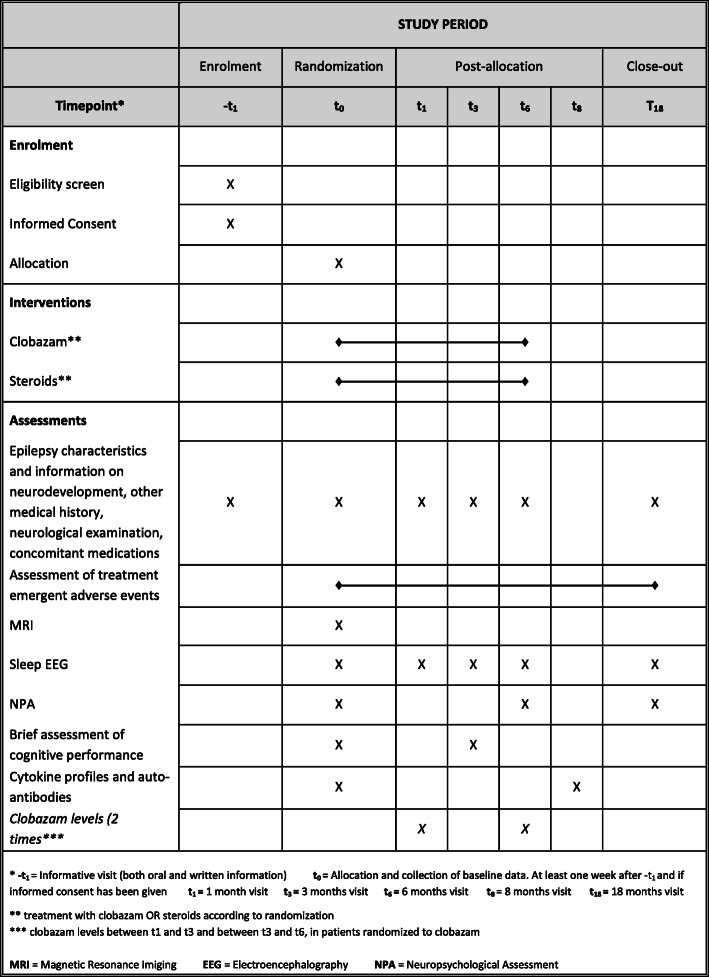
Schedule of enrolment, interventions, and assessments for participants

A schedule of enrolment, interventions, and assessments for participants is provided (Fig. 1).

### Statistical analysis

The primary analyses will be performed according to the intention-to-treat principle. We will also perform (secondary) analyses in patients who have completed their assigned treatment for the period of 6 months.

Continuous outcomes will be presented with means and 95% confidence intervals. These outcomes will primarily be compared between the two treatment groups using a *t* test or Mann-Whitney *U* test depending on the distribution of the data (normal vs not normal). Categorical outcomes will be presented as proportions with 95% confidence intervals. These outcomes will primarily be compared between the two treatment groups using Fisher’s exact test. Rates of (serious) adverse events will be compared in terms of risk ratios with corresponding 95% confidence intervals. We will also identify the proportion of patients that continue on the initially allocated treatment throughout the entire study period, and analyse possible predictors for discontinuation.

Although the stratified randomisation procedure intends to create two groups with equal patient characteristics, it is known that in randomised trials of < 1000 patients, there still is a risk of bias by chance [25]. Adjusted analyses are often performed to reduce the influence of the possible differences between the two treatment arms [26]. Therefore, possible predictors of treatment outcome (i.e. known prognostic factors) will be included in a multivariate logistic/linear regression model. These prognostic factors are as follows: age at ESES recording, time interval between ESES recording and inclusion, IQ levels and cognitive sum scores at enrolment, number of drugs administered before enrolment, and aetiology (unknown, structural, metabolic, genetic, immune, infectious). We will also include cytokine and autoantibody profiles as possible predictors.

The prospective design and structured follow-up schedule should minimise the amount of missing data. However, to account for possible influence of missing data, we will perform a sensitivity analysis using multiple imputation methods [27].

### Sample size calculation

A formal sample size calculation is hampered by the fact that no previous trials with these interventions have been performed. Our recently performed meta-analysis of published cases with epileptic encephalopathy with ESES provides the basis for estimating the difference in proportions of successfully treated patients that might be expected between corticosteroids and benzodiazepines. In our meta-analysis, treatment success was defined as improvement in EEG (at least 25% decrease in SWI) or cognition (10 IQ points or improvement defined by author). Differences in proportions were 25–30% between the two treatment groups in favour of corticosteroids. However, these results are of limited value because of the small sample size in the included studies and their mostly retrospective design.

In the RESCUE ESES study, we aim to include a total of 130 children, of whom 65 will be randomised to treatment with corticosteroids and 65 to clobazam. This sample size permits to detect a difference of 25% in the proportion of successfully treated patients between the two treatment arms (for example 50% vs 25%). In fact, 116 patients are needed to identify this difference with a power of 80% and a two-sided alpha of 5%, and we additionally account for a possible dropout rate of 10%.

As mentioned above, success is defined as improvement of 0.75 of the standard deviation of either IQ or the cognitive sum score. Differences of this magnitude have been reported in earlier observational (non-randomised) studies in this area [28]. Displaying the primary outcome as a dichotomous value (instead of a continuous value) will lead to a conservative estimation of the difference in effect. Therefore, our sample size calculation is likely to be an overestimation of the required number of patients for the continuous outcomes.

For continuous outcomes, we can detect standardised mean differences or Cohen’s *d* of around 0.5 (power = 80%, two-sided alpha of 5%). For Cohen’s *d*, an effect size of 0.2 to 0.3 is considered a “small” effect, around 0.5 a “medium” effect, and above 0.8 a “large” effect [29]. This means that “medium” effects on secondary outcomes can be detected by our trial including 130 children. Furthermore, as the primary outcome can also be displayed as a continuous variable (IQ or cognitive sum score), this also applies to analysis of the primary outcome as a continuous variable.

### Recruitment and promotion of participant retention

Because encephalopathy with ESES is relatively rare, we initiated a collaboration with European centres with a high level of expertise in epilepsy. The trial was announced at several international congresses, in newsletters of national and international paediatric neurology and epilepsy associations, and in patient magazines. Online as well as local training meetings for study personnel were organised. The study team is available for any questions or concerns from patients and their parents, and an independent physician is available for questions or complaints. If patients discontinue participation in the study or a protocol deviation occurs, the baseline and outcome measures that have been collected before discontinuation will still be analysed, e.g. if discontinuation occurred after 6 months of follow-up, the collected data will be included in primary outcome assessment. If they switch to the other treatment arm, but agree to continued follow-up according to the study protocol, their outcomes will still be analysed in the intention-to-treat analysis.

### Safety and tolerability

In accordance with legal requirements, the investigator will inform study participants and the reviewing accredited Ethics Committees (EC) if any data or findings emerge during the conduct of the study that suggest that risks involved in participation may outweigh potential benefits. Considering that both study medications have been widely prescribed for many years and their side effect profiles are well-known, the occurrence of suspected unexpected serious adverse reactions (SUSARs) is unlikely. The sponsor has a liability insurance which is in accordance with the European Clinical Trial directive 2001/20/EC. The sponsor also has an existing participant insurance which is in accordance with the legal requirements in the Netherlands. In all participating centres, a participant insurance has been contracted if there was no existing patient insurance that applies for the current study, unless an exemption of insurance requirements was possible considering the pragmatic design of the study, closely resembling current clinical practice.

Adverse events will be specifically asked for during the study visits, and parents of study participants will be instructed to contact their treating physician if any serious event will occur. In addition, general practitioners of participants will be informed of study participation and asked to contact the study team if any adverse events will be noted.

Adverse events will be reported according to Good Clinical Practice (GCP) guidelines. Adverse events are defined as any undesirable medical experience occurring to a subject during the study, whether or not considered related to the investigational drug. A serious adverse event (SAE) is any untoward medical occurrence or effect that at any dose results in death, is life-threatening, requires hospitalisation or extension thereof, results in persistent or significant disability or incapacity or otherwise jeopardises the subject, or requires intervention. SUSARs are SAEs that probably relate to the investigational drug and that are unexpected with regard to the drug used. If there is any uncertainty regarding relation between the study medication and a severe adverse event, this will be discussed within the steering committee of the trial. All adverse events are recorded and reported by the sponsor. SAEs will be reported to the ethics committee that approved the study within 15 days and followed up until they have abated or until a stable situation has been reached. SUSARs will be reported to the ethics committee and competent authority of the country where the SUSAR occurred within 15 days, and for fatal or life-threatening cases, a preliminary report will be sent within 7 days. The SUSAR will be reported to the EMA EudraVigilance database. A developmental safety update report (DSUR) will be provided annually to the ethics committees and competent authorities. An overview of adverse events per study treatment will be included in the final trial publication.

We will perform an interim analysis by the time primary outcome can be analysed in half of the required patients to evaluate whether there are unexpectedly large differences in effectiveness and unexpected side effects in both treatment groups. This analysis will form the basis to determine whether continuation of the trial is ethically justified.

### Monitoring

Because both treatments in this trial are also given as part of standard patient care, the study was classified as a (pragmatic) low-risk trial. A data safety and monitoring board (DSMB) was therefore not required. At the coordinating centre, UMC Utrecht, monitoring is performed by the hospital’s dedicated team of study monitors (not directly related to the study team). In the participating centres, monitoring is performed by clinical trial units affiliated with the European Clinical Research Infrastructure Network (ECRIN). An initiation visit, annual monitoring visits, and a close-out visit are performed in all centres in accordance with GCP guidelines. Monitoring procedures include a general control of the conduct and progress of the trial, study files, as well as a 100% check of the presence and correctness of informed consent forms, SAEs, and SUSARs as well as source data verification for 10% of collected data. A written monitor report will be provided by the monitor after each monitoring visit and includes a list of proposed measures and recommendations to ensure compliance with the study protocol.

## Discussion

Encephalopathy with ESES is characterised by sleep-induced epileptic activity accompanied by acquired cognitive deficits. Treatment aims at improving cognitive functioning. Current evidence regarding treatment efficacy is limited to mostly retrospective case series and indicates that conventional anti-epileptic drugs are often not effective, and that benzodiazepines and corticosteroids can provide greater benefit in improving cognition and EEG abnormalities. In patients with an operable structural lesion, surgery seems to be the most effective treatment.

The current study aims at comparing the efficacy of corticosteroids versus clobazam in improving cognitive functioning in children with ESES. The co-primary outcomes are cognitive functioning, as measured with total IQ scores and cognitive sum scores after 6 months. Secondary outcomes include cognitive functioning after 18 months, the spike wave index after 6 and 18 months, safety and tolerability, subjective assessment of daily functioning, and whether disease severity and treatment effect can be predicted, among other characteristics, by measuring serum levels of cytokines or autoantibodies.

The conduct of this multinational multicentre study in the rare population of patients with ESES is challenging for several reasons. Firstly, the differences in legislation and regulations between the involved countries and the requirement of obtaining ethics committee and competent authority approval for each of these countries are difficult and time-consuming. Secondly, although clobazam and corticosteroids are widely prescribed in regular clinical care, in some countries, labelling of the study drugs for this specific indication was considered to be required and posed a logistical challenge. Thirdly, each centre had their own requirements in terms of clinical trial contracts and this resulted in numerous email and telephone discussions between legal representatives. Altogether, these challenges caused considerable delay of the initiation of study at participating centres and resulted in withdrawal of several centres.

Despite the good intentions of local investigators, announcements on congresses, in newsletters, on websites and RESCUE ESES newsletters and promotional material, recruitment of patients in the centres where the study was initiated has so far been slower than expected. No general reasons have become apparent, but a few possible explanations have been proposed. Some patients did not participate because they considered the study investigations time-consuming and were concerned that their treatment might be delayed. Others had a strong preference for one of the treatment arms, e.g. because of the ease of use of clobazam or presumed higher effectivity of corticosteroids. Furthermore, the investigators involved are only supported in trial management and not financially compensated for time spent on study procedures. In addition, some local investigators mentioned that it was difficult to influence local referral patterns and that they failed to promote referral of additional ESES patients for enrolment in the study.

To conclude, we believe that this study addresses an important open question and that the results may guide clinicians in choosing the best treatment for patients with epileptic encephalopathy with ESES.

## Trial status

The study protocol (latest version: 11, 25 November 2014) was approved by ethics committees for conduct in the University Medical Center Utrecht in the Netherlands; University Hospital (UZ) Brussel and University Hospital (UZ) Leuven in Belgium; University Hospital Freiburg, Epilepsy Center Kork (Kehl), the Northern German Epilepsy Center Raisdorf, and Schön Klinik Vogtareuth in Germany; Filadelfia Epilepsy Hospital (Dianalund) in Denmark; Helsinki University Hospital in Finland; University Hospital Lyon (HCL), University Hospital Paris, and University Hospital Strasbourg in France; University Hospital San Carlos (Madrid) in Spain; and Great Ormond Street Hospital for Children (London), Royal Hospital for Sick Children Edinburgh, and Royal Hospital for Sick Children Glasgow in the UK. Approval by the ethics committee of the University Children’s Hospital Zurich, Switzerland, is expected soon. The first patient was included on 22 July 2014, and to date (2 April 2020), 43 patients have been included. The study end date is currently scheduled at 31 December 2020, but study extension will be considered.

## Supplementary Information

**Additional file 1.** SPIRIT 2013 Checklist: Recommended items to address in a clinical trial protocol and related documents*.

## Data Availability

Not applicable.
